# Impact of COVID-19 on routine malaria indicators in Uganda: An interrupted time series analysis

**DOI:** 10.21203/rs.3.rs-819495/v1

**Published:** 2021-08-18

**Authors:** Jane Frances Namuganga, Jessica Briggs, Michelle E Roh, Jaffer Okiring, Yasin Kisambira, Asadu Sserwanga, James Apollo Kapisi, Emmanuel Arinaitwe, Chris Ebong, Isaac Ssewanyana, Bryan Greenhouse, Catherine Maiteki-Ssebuguzi, Moses R Kamya, Sarah G Staedke, Grant Dorsey, Joaniter I Nankabirwa

**Affiliations:** Infectious Diseases Research Collaboration; University of California San Francisco; University of California San Francisco; IDRC: Infectious Diseases Research Collaboration; IDRC: Infectious Diseases Research Collaboration; Infectious Diseases Research Collaboration; Infectious Diseases Research Collaboration; Infectious Diseases Research Collaboration; Infectious Diseases Research Collaboration; IDRC: Infectious Diseases Research Collaboration; University of California San Francisco; MOH: Republic of Uganda Ministry of Health; IDRC: Infectious Diseases Research Collaboration; London School of Hygiene & Tropical Medicine; University of California San Francisco; Infectious Diseases Research Collaboration

**Keywords:** Infectious Diseases, COVID-19 pandemic, ITSA, Pre-COVID, rapid diagnostic tests (RDTs)

## Abstract

**Background:**

In March 2020, the government of Uganda implemented a strict lockdown policy in response to the COVID-19 pandemic. We performed an interrupted time series analysis (ITSA) to assess whether major changes in healthcare seeking behavior, malaria burden, and case management occurred after the onset of the COVID-19 epidemic.

**Methods:**

Individual level data from all outpatient visits occurring from April 2017 through March 2021 at 17 facilities were analyzed. Outcomes included total outpatient visits, malaria cases, non-malarial visits, proportion of visits with suspected malaria, proportion of patients tested using rapid diagnostic tests (RDTs), and proportion of malaria cases prescribed artemether-lumefantrine (AL). Pre-COVID trends measured over a three-year period were extrapolated into the post-COVID period (April 2020- March 2021) using Poisson regression with generalized estimating equations or fractional regression. Effects of COVID-19 were estimated over the 12-month post-COVID period by dividing observed values by the predicted values and expressed as ratios.

**Results:**

A total of 1,442,737 outpatient visits were recorded. Malaria was suspected in 55.3% of visits and 98.8% of these had a malaria diagnostic test performed. ITSA showed no differences in the observed versus predicted total outpatient visits, malaria cases, non-malarial visits, or proportion of visits with suspected malaria. However, in the second six months of the post-COVID period, there was a smaller mean proportion of patients tested with RDTs compared to predicted (Relative Prevalence Ratio (RPR) = 0.87, CI [0.78, 0.97]) and a smaller mean proportion of malaria cases prescribed AL (RPR = 0.94, CI [0.90, 0.99].

**Conclusions:**

There was evidence for a modest decrease in the proportion of RDTs used for malaria diagnosis and the proportion of patients prescribed AL in the second half of the post-COVID year, while other malaria indicators remained stable. Continued surveillance will be essential to monitor for changes in trends in malaria indicators so that Uganda can quickly and flexibly respond to challenges imposed by COVID-19.

## Introduction

Significant progress in malaria control has been realized in sub-Saharan Africa over the last two decades following the scale-up of effective malaria control interventions (1). At the start of the COVID-19 pandemic, there was concern that this progress in the fight against malaria would be reversed due to interruption of malaria control interventions and overwhelmed health care systems, with some modelling studies suggesting that malaria morbidity and/or mortality may double due to COVID-19 (2–4). In Uganda, malaria is endemic in over 95% of the country and the leading cause of morbidity and mortality, accounting for 30–50% of outpatient visits, 15–20% of all hospital admissions, and up to 20% of all hospital deaths (5). While successes have been registered in malaria control in the country in the last decade, including increased coverage of control interventions and reductions in the overall disease burden (6, 7), there is concern that these achievements may be reversed by the global COVID-19 pandemic.

The World Health Organization declared COVID-19 a global pandemic on 11 March 2020 (8). The Government of Uganda swiftly implemented strict restrictions and diverted personnel and resources to minimize the spread of SARS-CoV-2 and mitigate the economic burden of the pandemic. On 18th March 2020, mass gatherings were suspended, and a 14-day quarantine was imposed on all travelers arriving in Uganda (8). Subsequently, all schools were closed on 20th March 2020. Additional restrictions were implemented over the next two months including: 1) closure of borders except for cargo and goods; 2) suspension of public transport and restrictions on movement of private vehicle; 3) mandatory testing of truck drivers; and 4) a national lockdown and curfew from 7pm to 6:30am (8). This was followed by closure of all schools on 20th March 2020. When the country registered its first confirmed case on 21st March 2020, additional restrictions were implemented including: 1) closure of borders except for cargo and goods on 21st March 2020; 2) suspension of public transport and restrictions on movement of private vehicle on 25th March 2020; 3) mandatory testing of truck drivers on 10th April 2021; and 4) a national lockdown and curfew from 7pm to 6:30am on 30th March 2020, initially for 14 days but was extended to 26th May 2020 when the country started easing the restrictions (8). Despite these measures, Uganda’s COVID-19 cases progressively increased over the period with the country registering its first 100 confirmed cases on 6th May 2020, first death on 23rd July 2020, first 1,000 cases on 9th June 2020 and first wave peak in December 2020.

Although lockdowns have been shown to minimize the spread of COVID-19(9–11), they may have major repercussions on malaria treatment and prevention including decreased access to health care, interruption of service delivery, and disruption of delivery of malaria control interventions such as long-lasting insecticide treated net (LLIN) or indoor residual spraying (IRS) campaigns. In addition, an increase in the incidence of fever cases due to COVID-19 may lead to an increase in the number of suspected malaria cases presenting at health facilities, increasing demand for malaria diagnostics, and potentially leading to over diagnosis and overtreatment of malaria and/or a shortage of diagnostics. Indeed, a modelling study by the WHO predicted that in the ‘worst case’ scenario where LLINs campaigns were cancelled and access to effective antimalarial treatment was severely disrupted, malaria deaths could increase by > 200% in Uganda as result of the COVID-19 pandemic (12).

In this study, we assessed the effect of COVID-19 on malaria disease burden and case management at 17 public health facilities across Uganda using an interrupted time series analysis. Results from the study will improve our understanding of the effect of COVID-19 on malaria care in Uganda and in other countries with similar settings.

## Methods

### Health-facility based surveillance

**Figure 1:**
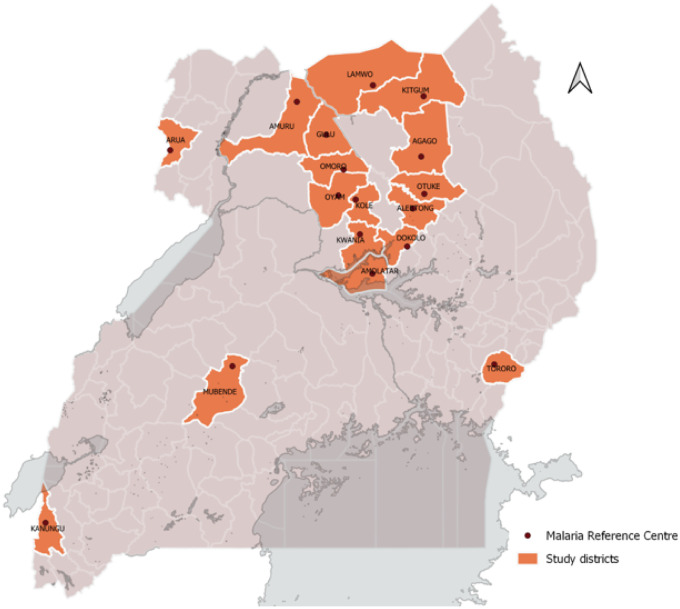
Map of Uganda with the 17 MRCs included in the analysis.

The study utilized data collected at 17 Malaria Reference Centers (MRCs) from April 2017 through March 2021 (Fig. 1). MRCs are high volume level III/IV public health facilities located throughout Uganda in areas with varying malaria transmission intensities. MRCs were first established in 2006 by the Uganda Malaria Surveillance Project (UMSP), a project led by Makerere University, University of California, San Francisco (UCSF), and Infectious Diseases Research Collaboration (IDRC) groups, in collaboration with the Uganda Ministry of Health. Details of the MRCs have been described elsewhere (13) but briefly, there are currently 70 MRCs collecting high quality individual level data from all patients presenting at the outpatient clinics of the facilities. Of the 70 MRCs, 17 were selected for this analysis based on the following criteria: 1) MRC was opened and data collection commenced before January 2017; and 2) MRC had less than 5% missing data on four of the variables (suspected malaria case, malaria test done, malaria test result recorded, and type of malaria test done).

At each MRC, patient data is recorded in standardized HMIS registers (HMIS 002 – outpatient register) at the outpatient clinics. Data is transcribed from the registers into Microsoft Access databases by on-site records officers. Data collected include patient demographics, village of residence, history of fever, whether a malaria diagnostic test was performed, type of malaria test done (rapid diagnostic test (RDT) versus microscopy), results of laboratory tests, diagnoses given, and treatments prescribed. Data is not collected on whether the patient receives or is adherent to the prescribed treatment. On average, over 1,400 patient visits and 1,000 suspected malaria cases tested are captured monthly at each MRC. Adherence to malaria case management guidelines is emphasized and supported at the MRCs through training, ongoing support from regional surveillance assistants, regular continuous medical education sessions, and monthly data analysis and feedback sessions for on-site health workers. Routine data quality assessments are undertaken at each site to ensure collection of high-quality data in terms of completeness and accuracy.

### Laboratory procedures

All patients suspected to have malaria are routinely sent to an onsite laboratory for confirmatory testing using either malaria microscopy or malaria RDT kits. Malaria microscopy at the MRCs is conducted by skilled laboratory personnel and generally reserved for when RDTs are not available or of limited stock and upon request from a clinician. Quality control for malaria microscopy is conducted monthly at each site by a laboratory team who are part of the core UMSP team. They also provide onsite mentorship for site laboratory personnel to ensure high quality malaria testing.

### Malaria control interventions

**Figure 2:**
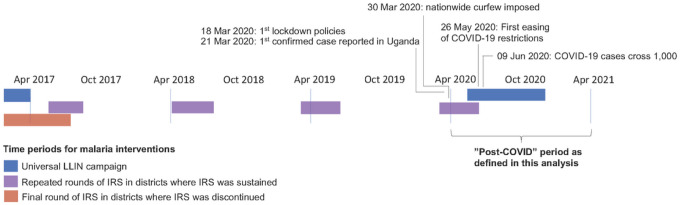
Timeline of malaria interventions and COVID-19 epidemic in Uganda.

Malaria control activities at the MRCs during the study period included: 1) malaria case management with artemether-lumefantrine (AL); 2) promotion of intermittent preventive treatment during pregnancy; and 3) universal distribution of free LLINs. Two national LLIN distribution campaigns were conducted during the study period, in 2017–18 and 2020–2021 (Fig. 2). The status of IRS campaigns varied across the sites included in this study as shown in Fig. 2; 3 sites were in districts that never received IRS, IRS was discontinued in 2017 in districts where 9 sites were located, and the remaining 5 sites were in districts that had sustained IRS campaigns throughout the study period.

### Statistical Analysis

Interrupted time series analyses (ITSA) were conducted to estimate the impact of the COVID-19 epidemic on key malaria indicators. Data from all 17 sites from April 2017 through the end of March 2021 were included in the analysis. The study period included 3 years “pre-COVID” and 1 year “post-COVID” with the start of the “post-COVID” period defined as April 1, 2020 (Fig. 2). Outcomes for the ITSA included number of total outpatient visits, number of non-malarial visits (visits in which malaria was not diagnosed), malaria visits (visits in which a laboratory-confirmed diagnosis of malaria was made, synonymous with malaria cases), proportion of all patient visits with suspected malaria, proportion of patients tested using RDTs, and proportion of patients with confirmed malaria prescribed AL.

To estimate the counterfactual trend, Poisson regression using a generalized estimating equation (GEE) with an autoregressive of order one correlation structure was used to model count outcomes; fractional regression was used to model proportional outcomes. All ITSA models included the following parameters: a linear term indicating the time in months since the start of the study observation period (models the pre-COVID trend); a linear term indicating the time in months since the start of the COVID epidemic (models the post-COVID trend), calendar month fixed effects (to model seasonality), and monthly rainfall data (mm) lagged by one month and averaged across the district-level. For ITSA models that included malaria outcomes, linear terms representing the number of months since LLIN distribution and the round of IRS were included to adjust for time-varying confounding. Based on surveillance data from these sites, LLIN campaigns were assumed to confer 24 months of protection, whereas IRS campaigns (which used Actellic or Sumishield-based insecticides) were assumed to provide up to a year of protection. For Poisson models, we assumed the underlying population was constant throughout the study period and thus no offset term was included.

Models were then used to predict counterfactual values for each health facility in each month (i.e., monthly number of events had the epidemic not occurred). For count outcomes, incidence rate ratios (IRR) were calculated by summing the outcomes across each post-policy period and dividing the observed by predicted values. For proportion outcomes, relative prevalence ratios (RPR) were calculated by averaging the monthly proportions across the post-policy period and dividing the observed by predicted (counterfactual) values. 95% confidence intervals were obtained using a 1000 block, percentile-bootstrapping procedure, where sites were resampled with replacement.

## Results

From April 2017 through March 2021, 1,442,737 patient visits were recorded at the 17 MRCs (Table 1). The median age of patients was 23 years, and the majority (67%) were female. Malaria was suspected in 798,270 (55.3%) of all patient visits, and almost all (98.8%) patients with suspected malaria had a malaria diagnostic test done. RDTs were the most common laboratory diagnostic tool used to test patients for malaria (83.8%), although this varied by site (range: 24.0–99.8%). Overall, malaria test positivity rate was 53.1%, ranging from 14.1–69.8% at individual MRCs. Most patients with confirmed malaria were prescribed AL (93%), the recommended first line treatment for uncomplicated malaria in Uganda. A detailed description of the study population stratified by site and status of IRS is presented in Table 1.

### Trends in disease burden and case management over the study period

**Figure 3:**
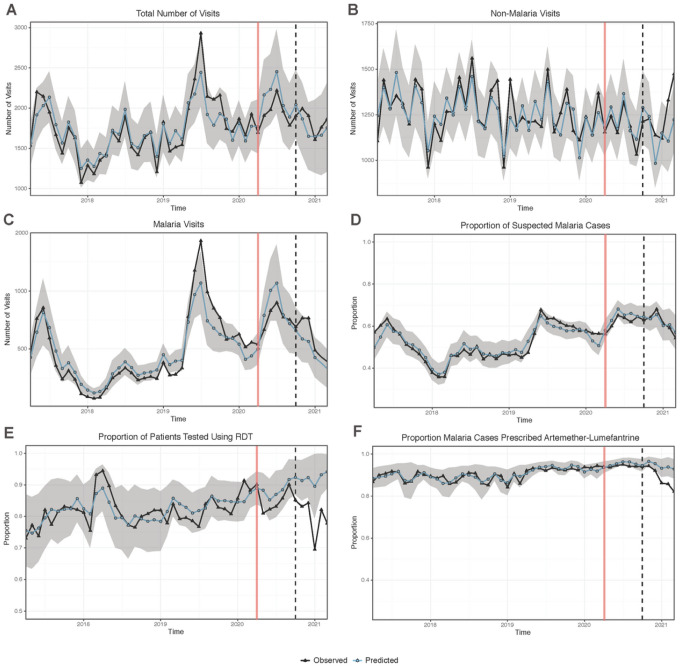
Predicted and observed A) total number of visits, B) non-malaria visits, C) visits where malaria was diagnosed, D) mean proportion of suspected malaria cases, and E) mean proportion of patients tested using RDT by month, and F) mean proportion of malaria cases prescribed artemether-lumefantrine. The grey ribbon represents the bootstrapped 95% confidence interval of the model. Vertical red line represents the start of the “post-COVID” period on April 1, 2020. Vertical black dashed line represents the 6-month midpoint of the post-lockdown year of data (October 1, 2020).

Over the four-year study period, a single annual peak was observed in the number of monthly outpatient visits, with the highest number of visits observed during mid-year of 2019 (> 2500 visits, Fig. 3A). The monthly trend in the number of outpatient visits for non-malarial illness was declining at a steady pace before the COVID-19 epidemic (Fig. 3B). The number of malaria visits followed a similar trend to that of the total outpatient visits, with peaks in these occurring simultaneously (Fig. 3C). Similar trends were observed with the proportion of suspected malaria cases (Fig. 3D). The proportion of patients tested with RDT each month remained consistently high (> 70%) and almost constant throughout the study period, except in 2018 when the proportion reached > 90% and in 2021 when the proportion fell below 70% (Fig. 3E). The proportion of confirmed malaria cases prescribed AL (Fig. 3F) was consistently high, remaining > 90% from late 2019 through 2020, before a decline was observed in 2021. The mean proportion of patients with suspected malaria who received a diagnostic test is not shown in Fig. 3 because it remained consistently high across all study time points (Supplemental Fig. 1) and therefore a formal ITSA was not performed.

### Impact of COVID-19 on disease burden and case management

Figure 3 shows that, for all outcomes except proportion of patients tested with RDT and proportion of malaria cases prescribed AL, there was no statistically significant change in the 12-month post-COVID period (April 2020 - March 2021); observed monthly values fall within the 95% confidence interval of the predicted values. This held true for total outpatient visits, malaria and non-malaria visits, and proportion of patients suspected to have malaria. However, for proportion of patients tested with RDTs and proportion of malaria cases prescribed AL, observed values were lower than expected in the last 6 months of observation based on pre-COVID trends. Effect estimates across all measured outcomes are presented in Table 2, stratified into the first and second six months post-COVID intervals. In the first six months after the onset of COVID-19 epidemic, during which the lockdown was strictest, there were no significant differences in the observed versus predicted numbers of total visits [196,300 vs 216,822; IRR = 0.91 (0.82–1.00)], malaria cases [75,825 vs 82,884; IRR = 0.92 (0.76–1.14)], and non-malarial visits [120,475 vs 123,902; IRR = 0.97 (0.92–1.04)]. There were also no significant differences in the mean proportion of suspected malaria cases, mean proportion tested with RDT, or mean proportion prescribed AL.

Over the second six months of the post-COVID period (October 2020-March 2021), during which lockdown restrictions were eased despite increasing COVID-19 cases in the country, there were again no significant differences observed versus predicted numbers total visits [187,573 vs 186,136; RR 1.01 (0.81–1.23)], malaria cases [60,032 vs 50,050, RR 1.20 (0.75, 1.84)] and non-malarial visits [127,541 vs 118,890; RR 1.07 (0.95–1.21)]. There was also no difference in the proportion of suspected malaria cases. However, for the mean proportion of patients tested with RDTs, there was a significantly smaller mean proportion of patients tested with RDTs compared to predicted [80.3% vs 92.2%; RR 0.87 (0.78, 0.97)]. In addition, there was a smaller mean proportion of malaria cases prescribed AL compared to predicted, [89.1% vs 94.4%; RPR = 0.94 (0.90, 0.99)]. This was likely due to an increase in the percentage of patients with laboratory confirmed malaria that received a prescription of dihydroartemisinin-piperaquine; this percentage rose from 0.04% in the pre-COVID period to 0.3% from April 2020-September 2020 and to 3.7% in the period from October 2020 through March 2021.

## Discussion

This study evaluated the impact of the COVID-19 pandemic on the number of outpatient visits, malaria disease burden, and case management practices between April 2020 and March 2021 using routine surveillance data collected at 17 high volume public outpatient facilities in areas of varying malaria transmission across Uganda. In this study the number of outpatient visits, malaria cases, and non-malaria visits as well as most indicators of malaria case management did not reflect any significant changes in the first year following the onset of the COVID-19 epidemic. However, from October 2020 through March 2021, a modestly lower mean proportion of suspected malaria patients received an RDT for malaria diagnosis compared to what was predicted for that period. Notably, this difference was not reflected by a change in the proportion of patients with suspected malaria who underwent diagnostic testing, as microscopy was available as an alternative to RDTs. A similar trend was observed in the last six months of observation for the mean proportion of malaria cases prescribed AL.

The emergence and rapid spread of COVID-19 across the world has created massive global disruptions on health systems, social services, and economic activity (14). The disruption in health services is expected to be magnified in sub-Saharan Africa to a greater degree than in other regions due to relatively weak health service infrastructures, low clinician to population ratios, limited laboratory capacity, and a higher burden of other infectious diseases (15). Delivery of malaria preventive measures and care services are some of the activities most likely to be affected by the epidemic given the high prevalence of malaria in the region (16). Indeed, there are predictions that malaria cases will increase, and malaria related mortality may nearly double, decelerating any gains attained in the last decade (3, 12, 17, 18). Uganda, one of the highest malaria burden countries in sub-Saharan Africa, was predicted to be one of the countries most likely to be affected by the COVID-19 pandemic. According to a WHO modelling study, by the end of 2020 in a scenario where LLIN campaigns are not implemented and LLINs continuous distributions and access to effective antimalarial treatment are reduced by up to 75% malaria deaths could increase by 200% in Uganda (12). A study by Bell et. al. predicted less disastrous effects resulting from a widespread COVID-19 outbreak compared to the impact of lockdowns on malaria programs (Bell, 2020).

However, contrary to those worst-case scenarios, these data suggest that despite travel restrictions and other lockdown measures, access to care and malaria case management were largely unaffected by the COVID-19 pandemic in the first year after it was confirmed in Uganda. This could be because 75% of the population in Uganda resides in rural areas where patients seeking care either walk or ride bicycles to the health facilities (19), making access to care possible even with restrictions to motor vehicle movements. In addition, the health system is structured in such a way that most of the health care staff are housed at or near the facilities; furthermore, travel permits were provided for health workers who needed to travel long distances to their duty stations, preventing the staff shortages that would likely have limited access to malaria care. Finally, planned malaria control activities, including the mass LLIN distribution campaign (implemented between June 2020 and March 2021) and IRS rounds, were successfully implemented despite the pandemic.

In addition, the outbreak was expected to increase fever cases because COVID-19 and malaria both present with fevers. However, community transmission of COVID-19 remained low during the first year following the outbreak; this may explain the fact that this analysis showed no increase in non-malarial visits or proportion of cases with suspected malaria. However, a decrease in the proportion of patients tested for malaria using RDTs was observed. This may be because of global supply chain issues that resulted in local stock outs of RDTs. In the last year, there was a noted global decline in malaria RDT production and supply, with manufactures shifting in focus from RDTs to COVID-19 point of care tests (20, 21). Importantly, the decrease in proportion of RDTs used as the laboratory diagnostic did not affect whether or not patients received a diagnostic test for suspected malaria, because microscopy was available as an alternative diagnostic at the MRCs.

There was also a modest decrease in the mean proportion of malaria cases prescribed AL, the standard of care for the treatment of uncomplicated malaria in Uganda, seen in the last six months of observation. Uganda’s Ministry of Health secured funding to procure dihydroartemisinin-piperaquine (DP) for use as an alternative first line treatment for uncomplicated malaria in the event of treatment failure for patients using AL and for prescription to all patients with severe malaria following completion of three intravenous artesunate doses. From 2020 to-date, DP has been available at some public health facilities for use as an alternative first line treatment for patients with treatment failure due to AL and all patients diagnosed with severe malaria following completion of artesunate treatment, leading to an increase in the number of patients prescribed DP. Therefore, the decrease in prescriptions of AL observed in the last six months of the study may not be due to a decrease in supply or availability of AL due to the COVID-19 pandemic but instead due to changing prescription practices in response to policy changes regarding DP. There was no direct data on AL stock captured at these health facilities over the timeframe of the analysis, as such we were unable to conclusively attribute this decline to AL stock-outs at the facilities.

Other studies in similarly resource-restricted settings have shown varied impacts of the COVID-19 pandemic on malaria burden and deaths. A brief report published regarding patients at one health facility in Sierra Leone showed a decrease in the number of malaria cases diagnosed in children younger than 5 years of age in April 2020 compared to April 2019, but this had recovered by May 2020 (22). However, this study was at a single health facility, did not account for seasonality, and only observed three months of the post-COVID period. A larger study in Zimbabwe, which utilized routine malaria surveillance data collected from all public and private health facilities from January 2017 to June 2020, found an excess of over 30,000 malaria cases from January to June 2020 compared to an average over the same period from 2017, 2018, and 2019 data (23). This study also found that the number of malaria deaths recorded from January to June 2020 exceeded the annual totals for 2018 and 2019. However, while they describe these increases by comparing numbers from January 2020 to June 2020 to prior data, they do not make any statistical inference regarding their findings and note the limitations of routine surveillance data regarding data quality. A particular strength of the study presented here is the use of a quasi-experimental, interrupted time series study design which accounted for pre-COVID trends, other factors such as seasonality, rainfall, differences in study site, and concurrent LLIN and IRS campaigns to predict outcomes one year after the onset of COVID-19. In addition, the MRCs included had a full three years of pre-COVID data that had undergone a supervised data cleaning process, resulting in less than 5% missing data.

Several limitations of this study are worth noting. First, the impact of the COVID-19 pandemic on malaria mortality was not assessed, as these data were not available. Second, data was only available on antimalarial prescription practices and not whether antimalarial drugs were actually available and administered. Third, COVID-19 cases were still relatively low in Uganda during the period this study was conducted, and it is likely that the impact of the COVID-19 pandemic on malaria indicators may change as community transmission increases, especially with the Delta variant now in the country. This is already being observed as the number of cases observed between March and July 2021 has doubled compared to what was observed between March and July 2020 (90,656 versus 40,962) (24). Thus, conclusions only reflect the effect of the COVID-19 on malaria indicators in the first year after arrival of COVID-19 in Uganda. Notably, COVID-19 is on the rise in Uganda and the Government of Uganda has imposed a second lockdown in June 2021. Further study on the impact of the epidemic and lockdown policy is needed. Fourth, this study makes use of high-quality data from 17 MRCs across varied transmission settings in Uganda, which may not be representative of the entire country. Though similar outcomes are available through national health management information systems (HMIS) databases, HMIS data have been shown to suffer from increased ‘missingness’ and quality control problems(25, 26) and to provide robust estimates these data were not included. Fifth, though ITSAs are known to be a robust method of utilizing longitudinal data for estimating causal effects (27, 28), we cannot rule out that estimates may have been subject to unmeasured confounding.

## Conclusion

Utilizing routine surveillance data from 17 health facilities across Uganda, this study found the onset of the COVID-19 epidemic did not have major effects on malaria disease burden and indicators of case management, except for a modest decrease in the mean proportion of RDTs used for malaria diagnosis and the mean proportion of malaria cases prescribed AL in the second half of the post-COVID year. However, continued surveillance will be essential to monitor for any changes in trends in malaria indicators due to the indirect effects of the COVID-19 pandemic so that necessary public health interventions can be instituted early to prevent increases in malaria morbidity and mortality. For example, now that a decrease in the usage of RDTs has been shown, it will be important to further investigate the cause of this decline and implement solutions by sourcing additional RDT supplies or ensuring access to high quality microscopy at all public health facilities.

## Supplementary Material

Supplement 1

## Figures and Tables

**Table 1:** Cumulative totals of key malaria indicators stratified by malaria reference center (MRC) from April 2017 through March 2021

IRS status	MRC	Total number of outpatient visits	Median age in years	Proportion female	Malaria suspected (% total visits)	Tested for malaria (% malaria suspected)	RDT performed (% tested for malaria)	Laboratory confirmed malaria (% tested for malaria)	AL prescribed* (% laboratory confirmed malaria)
No recent history	Kasambya	59,574	20.7	67.5	44,118 (74.1)	42,830 (97.1)	24,522 (57.3)	19,100 (44.6)	18,801 (98.4)
	Kihihi	79,561	25.2	65.6	46,207 (58.1)	46,153 (99.9)	11,061 (24.0)	17,322 (37.5)	16,099 (92.9)
	Opia	57,879	17.7	63.6	46,586 (80.5)	46,335 (99.5)	46,270 (99.8)	28,378 (61.2)	28,091 (99.0)
IRS discontinued	Aduku	102,761	24.7	69.9	58,404 (56.8)	57,157 (97.9)	36,702 (64.2)	31,723 (55.5)	30,910 (97.4)
	Anyeke	108,612	24.6	66.3	52,820 (48.6)	51,765 (98.0)	46,143 (89.1)	35,056 (67.7)	32,089 (91.5)
	Aboke	81,626	24.2	68.6	50,075 (61.3)	49,740 (99.3)	45,908 (92.3)	34,696 (69.8)	32,920 (94.9)
	Awach	110,328	20.7	69.2	61,252 (55.5)	59,357 (96.9)	58,183 (98.0)	38,343 (64.6)	35,956 (93.8)
	Lalogi	110,585	21.8	68.7	67,335 (60.9)	67,095 (99.6)	65,652 (97.8)	38,572 (57.5)	35,315 (91.6)
	Patongo	77,556	20.4	66.2	54,394 (70.1)	53,794 (98.9)	51,244 (95.3)	29,858 (55.5)	28,619 (95.9)
	Atiak	87,872	19.8	65.5	49,529 (56.4)	48,685 (98.3)	48,477 (99.6)	32,894 (67.6)	30,530 (92.8)
	Padibe	89.262	21.3	66.5	56,985 (63.8)	56,849 (99.8)	55,864 (98.3)	35,023 (61.6)	32,484 (92.8)
	Namokora	92,605	20.6	65.0	64,242 (69.4)	63,930 (99.5)	57,983 (90.7)	36,163 (56.5)	32,216 (89.2)
IRS sustained	Nagongera	77,735	23.3	65.8	27,667 (35.6)	27,623 (99.8)	14,286 (51.7)	3,891 (14.1)	3,459 (88.9)
	Amolatar	71,440	26.5	65.8	19,231 (26.9)	19,132 (99.5)	16,302 (85.2)	6173 (32.3)	5417 (87.8)
	Dokolo	115,297	27.1	68.6	42,046 (36.5)	41,847 (99.5)	38,555 (92.1)	13,124 (31.4)	11,617 (88.5)
	Orum	47,440	26.5	65.5	26,265 (55.4)	26,252 (100)	16,128 (61.4)	10,162 (38.7)	7,799 (76.7)
	Alebtong	72,604	27.8	70.1	31,114 (42.9)	30,424 (97.8)	28,173 (92.6)	8,379 (27.5)	7,726 (92.2)
All sites combined		1,442,737	23.0	67.0	798,270 (55.3)	788,988 (98.8)	661,453 (83.8)	418,830 (53.1)	390,048 (93.1)

* Only includes those with laboratory confirmed malaria

**Table 2:** Estimates of the impact of COVID-19 epidemic on disease burden during the first and second six months “post-COVID”

Outcomes	April 2020 – September 2020			October 2020 – March 2021		
	Observed^a^	Predicted^a^	Ratio [95% CI]^b^	Observed^a^	Predicted^a^	Ratio [95% CI]^b^
Total number of outpatient visits	196,300	216,822	0.91 [0.82, 1.00]	187,573	186,136	1.01 [0.81,1.23]
No of visits with a malaria diagnosis	75,825	82,8842	0.92 [0.76, 1.14]	60,032	50,050	1.20 [0.75, 1.84]
Number of non-malaria visits	120,475	123,902	0.97 [0.92, 1.04]	127,541	118,890	1.07 [0.95, 1.21]
% Suspected/Visits	61.9%	64.1%	0.97 [0.93, 1.04]	61.6%	61.9%	0.99 [0.89,1.22]
% Tested with RDT	85.4%	88.3%	0.97 [0.91, 1.01]	80.3%	92.2%	0.87 [0.78,0.97]
% Malaria cases prescribed AL	94.2%	95.2%	0.99 [0.98, 1.00]	89.1%	94.4%	0.94 [0.90, 0.99]

a Estimates reported as the total number of visits or mean proportion during the 1-year post-policy period.

b For visit outcomes, this represents an incidence rate ratio; for proportions, a relative percent ratio. Assumed a constant population at-risk over the study period

## Data Availability

The datasets generated and/or analyzed during the current study are available at https://github.com/EPPIcenter/COVID_ITSA
